# Gram-negative Organisms from Patients with Community-Acquired Urinary Tract Infections and Associated Risk Factors for Antimicrobial Resistance: A Single-Center Retrospective Observational Study in Japan

**DOI:** 10.3390/antibiotics9080438

**Published:** 2020-07-23

**Authors:** Naoki Kanda, Hideki Hashimoto, Tomohiro Sonoo, Hiromu Naraba, Yuji Takahashi, Kensuke Nakamura, Shuji Hatakeyama

**Affiliations:** 1 Division of General Internal Medicine, Jichi Medical University Hospital, Tochigi 329-0498, Japan; shatake-tky@umin.ac.jp; 2 Department of Emergency and Critical Care Medicine, Hitachi General Hospital, Ibaraki 317-0077, Japan; hidehashimoto-tky@umin.ac.jp (H.H.); sonopy77@gmail.com (T.S.); nrbhrm@gmail.com (H.N.); yuji.mail@icloud.com (Y.T.); mamashockpapashock@yahoo.co.jp (K.N.)

**Keywords:** urinary tract infection, antibiotic resistance, antibiogram, nursing home, empiric therapy

## Abstract

A specific antibiogram is necessary for the empiric antibiotic treatment of community-acquired urinary tract infections (UTI) because of the global spread of antimicrobial resistance. This study aimed to develop an antibiogram specific for community-acquired UTI and assess the risk factors associated with community-acquired UTI caused by antimicrobial-resistant organisms. This cross-sectional observational retrospective study included patients with community-acquired UTI caused by Gram-negative rods (GNR) who were admitted to the emergency department at a tertiary care hospital in Ibaraki, Japan, in 2017–2018. A total of 172 patients were enrolled (including 38 nursing home residents). Of the 181 GNR strains considered as causative agents, 135 (75%) were *Escherichia coli*, and 40 (22%) exhibited third-generation cephalosporin resistance. Extended-spectrum β-lactamase (ESBL)-producing *E. coli* accounted for 25/40 (63%) of resistant GNR. Overall susceptibility rate of Enterobacterales was 92%, 81%, 100%, 75%, and 89% for cefmetazole, ceftriaxone, meropenem, levofloxacin, and trimethoprim–sulfamethoxazole, respectively. Residence in a nursing home (odds ratio (OR), 2.83; 95% confidence interval (CI), 1.18–6.79) and recent antibiotic use (OR, 4.52; 95% CI, 1.02–19.97) were independent risk factors for UTI with resistant GNR. ESBL-producing *E. coli* was revealed to have a strong impact on antimicrobial resistance pattern. Therefore, an antibiotic strategy based on a disease-specific antibiogram is required.

## 1. Introduction

Urinary tract infection (UTI) is a common infectious disease worldwide [1,2,3]. Lower UTIs such as cystitis are usually treated in the outpatient setting. However, upper UTIs such as pyelonephritis or kidney abscess are often complicated by sepsis and/or bacteremia [4]; therefore, intravenous empiric antibiotic therapy and hospitalization are frequently required. UTIs are caused by several different types of bacteria, so-called uropathogens. While *Escherichia coli* is the most frequent causative agent, other Enterobacterales, enterococci, and *Pseudomonas aeruginosa* also cause UTIs.

Over the past decade, antimicrobial resistance has become a global threat [4,5,6]. The mechanisms of β-lactam resistance in Enterobacterales include extended-spectrum β-lactamases (ESBL), AmpC, and carbapenemases, such as *Klebsiella pneumoniae* carbapenemase (KPC) or metallo-β-lactamase [7,8]. Enterobacterales possessing β-lactam resistance often pose resistance to other classes of antibiotics [9,10]. Although the prevalence of carbapenemase-producing Enterobacterales (CPE) is very limited in Japan so far, the prevalence of ESBL-producing Enterobacterales has been increasing in recent years. According to a report by the Japan Nosocomial Infections Surveillance (JANIS) system, the susceptibility rates to cefotaxime and levofloxacin among *E. coli* isolates were 72% and 58%, respectively, in 2017, compared to 92% and 75%, respectively, in 2007 [11].

The association between antimicrobial resistance and patient risk factors has been investigated in several studies [12,13,14,15]. A retrospective multivariate analysis performed by a Chicago emergency department showed that multidrug-resistant Enterobacteriaceae infections were associated with prior fluoroquinolone use, healthcare exposure, and presence of obstructive uropathy [12]. Another study from Spain indicated that healthcare-associated UTI and antibiotic use in the previous month were independent risk factors for fluoroquinolone resistance [13].

In addition to the risk factors, local and/or regional antibiogram data should be considered before making a decision regarding initial empiric antibiotic therapy for UTI. In general, a hospital antibiogram is constructed by the clinical microbiology laboratory using hospital-wide microbiology data, including all culture results collected in the outpatient and inpatient settings, regardless of whether the isolated bacteria cause infections or not. Therefore, the surveillance of microbiology data and antibiogram of a specific infection along with patient information is desirable.

We conducted a retrospective observational study of the antimicrobial resistance pattern of Gram-negative rods (GNR) causing UTIs in recent years. Furthermore, we analyzed the association between antimicrobial resistance and patient risk factors. The aim of this study was to assess the optimal empiric antibiotic therapy for community-acquired UTI based on specific microbiology and antibiogram data.

## 2. Methods

### 2.1. Study Design

This was a cross-sectional observational study utilizing an existing database and hospital records at Hitachi General Hospital, a tertiary care 651-bed hospital in Ibaraki Prefecture, Japan. Our database included the diagnoses and clinical assessments of all patients admitted to the emergency and intensive care departments. We defined the Gram-negative bacteria resistant to ceftriaxone as resistant GNR, and analyzed the risk factors for the detection of resistant GNR in patients with community-acquired UTI. This study was approved by the Ethics Committee of the Hitachi General Hospital (Number 2017-95). The requirement for written informed consent from the enrolled patients was waived by the Ethics Committee because of the retrospective design of the study.

### 2.2. Study Population

The database used in this study contained the data of all patients admitted to our department from 1 January 2017 to 31 December 2018. The records of all the patients who were diagnosed with UTI, including pyelonephritis, kidney abscess, prostatitis, and prostate abscess, were collected. We also analyzed the urine and blood culture results of each patient using microbiological data from the hospital microbiology laboratory. Subsequently, we included adult patients who were diagnosed with community-acquired UTI caused by GNR in our analysis. If a patient had multiple UTI episodes in the study period, we included only the first episode.

### 2.3. Data Collection

Clinical information extracted from the database included patient age, sex, residence in nursing home, antibiotic use within the last three months, hospital admission within the last three months, history of isolation of resistant GNR within the last six months, bed-ridden status (unable to get off the bed without assistance), comorbidities (including diabetes mellitus, malignancy, and immunodeficiency), and placement of a long-term urinary catheter before admission.

The automated Vitek 2 (bioMerieux) method was used for bacterial identification and antimicrobial susceptibility testing of GNRs. Susceptibility test results were interpreted according to the Clinical and Laboratory Standards Institute (CLSI) breakpoints [16]. The third-generation cephalosporin used for susceptibility testing was changed from ceftriaxone to cefotaxime in our hospital during the study period. Therefore, we have presented the susceptibility results for cefotaxime instead of ceftriaxone throughout this manuscript. The ESBL confirmation tests were performed using the MicroScan Panel (Beckman Coulter). When multiple GNRs were isolated from a single patient, we regarded the patient as being in the resistant GNR group if at least one GNR was resistant to ceftriaxone.

### 2.4. Statistical Analysis

We analyzed the prevalence of pathogens, the rate of ESBL-producing organisms, and susceptibility rates to several antibiotics including ampicillin, ampicillin-sulbactam, piperacillin-tazobactam, cefazolin, cefmetazole, ceftriaxone, cefotaxime, cefepime, meropenem, aztreonam, amikacin, levofloxacin, trimethoprim-sulfamethoxazole (TMP-SMX), and minocycline. Univariate analysis was conducted by Pearson’s chi-square test or Fisher’s exact test, as appropriate, for categorical variables, or the Mann–Whitney U test for continuous variables. Multivariate logistic regression was performed to identify the independent risk factors for resistant GNR. Age, residence in nursing home, prior antibiotic use, and long-term urinary catheter placement were included as variables based on the evidence from previous studies [10,12,13]. The robustness of the model was confirmed by the Hosmer–Lemeshow goodness-of-fit statistic. All *p*-values were 2-tailed; *p*-values less than 0.05 were defined as statistically significant. All statistical analyses were performed using SPSS (version 22.0, SPSS Inc., Chicago, IL, USA).

## 3. Results

Among 4328 patients who were admitted to our department during the study period, 271 were diagnosed with UTI. Of these, 48 patients had hospital-associated UTI, 22 had UTI caused by non-GNR bacteria, 16 had UTI without pathogen identification, and 13 had a recurrent episode of UTI and were excluded from the study. Finally, 172 patients who had community-acquired UTI caused by GNR were included in the analyses. A total of 181 strains of GNR that were considered as causative pathogens were identified. Among them, 40 resistant GNR strains were isolated from 37 patients, and were classified as the resistant GNR group.

Patient characteristics are shown in Table 1. The median age was 80 years, 60 patients (35%) were men, and 81 (47%) cases were complicated with bacteremia. The average length of stay in the intensive care and/or high care units was 3.6 days. Pyelonephritis accounted for 96% (165/172) of the UTIs, and the rest comprised three kidney abscesses, three prostatitis cases, and one prostate abscess. Nursing home residency, preceding antibiotic use, recent hospital admission, resistant GNR colonization, bed-ridden status, and long-term urinary catheter placement were significantly more frequently observed in the resistant GNR group than in the non-resistant GNR group.

Table 2 shows the microbiology, prevalence of resistant GNR and ESBL-producing bacteria, and susceptibility rates for antibiotics. Of the 181 GNR strains, *E. coli* accounted for 75% (135/181), *Klebsiella* spp. for 12% (22/181), and *P. aeruginosa* for 4% (8/181). ESBL-producing GNR comprised 65% (26/40) of resistant GNR, and all but one were ESBL-producing *E. coli* (25/26). The overall susceptibility rate of Enterobacterales in this study was 68% for ampicillin-sulbactam, 99% for piperacillin-tazobactam, 92% for cefmetazole, 81% for ceftriaxone, 83% for cefepime, 100% for meropenem, 75% for levofloxacin, 89% for TMP-SMX, and 87% for minocycline (Table 3).

Multivariate logistic analysis revealed that nursing home residence (odds ratio (OR), 2.83; 95% confidence interval (CI), 1.18–6.79) and antibiotic use within the last three months (OR, 4.52; 95% CI, 1.02–19.97) were independent risk factors for community-acquired UTI caused by resistant GNRs (Table 4). The susceptibility rate of Enterobacterales among patients living in nursing homes was 100% for piperacillin-tazobactam, 90% for cefmetazole, 64% for ceftriaxone, 69% for cefepime, 54% for levofloxacin, 79% for TMP-SMX, and 82% for minocycline (Table 3).

## 4. Discussion

Our study investigated the etiology, risk factors, and antibiotic resistance pattern of GNRs causing community-acquired UTI. The most frequently isolated species from community-acquired UTI was *E. coli*, which comprised the majority of antibiotic resistant GNRs. There have been several studies regarding the increasing rate of resistant GNRs globally [9,10,17,18,19]. The ESBL-producing rate among *E. coli* isolated from patients with UTI increased from 10.4% to 13.0% in Canada, and from 7.8% to 18.3% in the US between 2010 and 2014 [10]. Although CPE are not prevalent in Japan yet, they have become an increasing threat worldwide [20,21]. CPE are mainly isolated from nosocomial specimens, but in one report, about 15% of the specimens that tested positive for CPE were from nursing home residents [22].

According to the JANIS system, the susceptibility rates for cefotaxime and levofloxacin among *E. coli* in 2017 were 72% and 58%, respectively, in Japan, and 73% and 62%, respectively, in Ibaraki Prefecture. As the JANIS system predominantly covers the isolates from hospitalized patients, the antibiotic susceptibility rates were considered to be lower than those from community-acquired specimens. In our study, the *E. coli* susceptibility rates for third generation cephalosporins (i.e., ceftriaxone and cefotaxime) and levofloxacin were 78% and 68%, respectively, suggesting that the susceptibility pattern of *E. coli* strains from patients with community-acquired UTI were similar to, or only 5–10% better than those from hospital-acquired infections.

We identified nursing home residence and prior antibiotic use within the last three months as independent risk factors associated with resistant GNR. These findings were consistent with those of previous studies in other countries [12,13,23]. In our study, long-term urinary catheter placement showed only a modest association (OR 2.77, *p* = 0.103) with the risk of resistant GNRs, which was not statistically significant. This may be partly attributed to the sample size of this study, which was insufficient for detecting a significant difference.

Several guidelines have emphasized the importance of taking an antibiogram into account when determining empiric treatment of UTIs [24,25,26,27]. In this study, the majority (95%) of the isolates from community-acquired UTI were Enterobacterales, and their overall susceptibility rates for ceftriaxone/cefotaxime and levofloxacin were almost 70–80%. Moreover, in terms of isolates from patients living in nursing homes, the susceptibility rate decreased to approximately 40%, mainly due to the prevalence of ESBL-producing *E. coli* strains. Optimal empiric antibiotic treatment options are limited; only piperacillin-tazobactam, cefmetazole, meropenem, and amikacin had a susceptibility rate of more than 90% to Enterobacterales in community-acquired UTI in this study.

Piperacillin-tazobactam and meropenem might be the reasonable choices for empiric antibiotic treatment in this setting. However, routine use of such broad-spectrum agents for community-acquired UTI may lead to further increases in antimicrobial resistance. A prospective study has demonstrated that piperacillin-tazobactam was inferior to carbapenems for the initial empiric treatment of bacteremia due to ESBL-producing Enterobacterales [28]. Cefmetazole may be an alternative option for the treatment of UTIs caused by ESBL-producing organisms, although only limited data is currently available [29,30].

There are several limitations to this study. First, this was a single-center retrospective study. Our sample size was relatively small; thus, the susceptibility pattern observed might not accurately represent the local epidemiology, particularly for organisms with less than 10 isolates. We collected patient information by performing a chart review, which may result in an information bias and some factors might be underestimated. In addition, selection bias could not be ruled out because this study was conducted at an emergency department. Therefore, more severely ill patients were likely to be included. Second, patients with false-negative urine cultures due to preceding antibiotic use were excluded from this study, although there were only a small number of such cases.

## 5. Conclusions

In conclusion, this observational study developed an antibiogram specific for GNRs from community-acquired UTIs and demonstrated that the patients who live in nursing homes and have a recent history of antibiotic use are at significant risk of community-acquired UTI caused by resistant GNRs. As ESBL-producing *E. coli* accounted for the majority of resistant GNRs, cefmetazole, piperacillin-tazobactam, and carbapenems may be reasonable options for empiric treatment, particularly among nursing home residents.

## Figures and Tables

**Table 1:** Patient characteristics.

Characteristics	Overall (*n* = 172)	Resistant GNR Group (*n* = 37)	Non-Resistant GNR Group (*n* = 135)	*p*-value
Age, median (IQR), years	80 (72–85)	80 (70–85)	79 (73–85)	0.906
Males, no. (%)	60 (35)	18 (49)	42 (31)	0.047
Length of ICU/HCU stay, mean (SD), days	3.6 (2.2)	3.8 (2.7)	3.5 (2.1)	0.481
Pyelonephritis, no. (%)	165 (96)	36 (97)	129 (96)	0.995
Bacteremia, no. (%)	81 (47)	16 (43)	65 (48)	0.596
Risk factors, no. (%)				
Nursing home residence	38 (22)	15 (41)	23 (17)	0.002
Antibiotic use within last 3 months	9 (5.2)	5 (14)	4 (3)	0.023
Hospitalization within last 3 months	10 (5.8)	6 (16)	4 (3)	0.007
Resistant GNR colonization ^a^	5 (2.9)	3 (8.1)	2 (1.5)	0.071
Bed-ridden status ^b^	30 (17)	13 (35)	17 (13)	0.001
Diabetes	47 (27)	8 (22)	39 (29)	0.415
Long-term urinary catheter	14 (8.1)	7 (19)	7 (5.2)	0.013
Immunosuppression ^c^	42 (24)	11 (30)	31 (23)	0.396

Abbreviations: IQR, interquartile range; GNR, Gram-negative rod; ICU, intensive care unit; HCU, high care unit; SD, standard deviation. Significant *p*-values are indicated in bold. ^a^ A history of positive resistant GNR culture within the last 6 months. ^b^ Patient unable to get off the bed without assistance. ^c^ Patient with malignancy or immunodeficiency and history of receiving immunosuppressive agents.

**Table 2:** Gram-negative rods causing urinary tract infection and their antimicrobial resistance pattern.

Factors	Total	*Escherichia Coli*	*Klebsiella* Spp.	*Pseudomonas Aeruginosa*	*Proteus Mirabilis*	*Enterobacter Cloacae*	*Providencia Rettgeri*	*Seratia Marcescens*	*Citrobacter* Spp.	Others
GNR no. (%)	181	135 (75)	22 (12)	8 (4.4)	4 (2.2)	3 (1.7)	3 (1.7)	2 (1.1)	2 (1.1)	2 (1.1)
Resistant GNR, no. (%)	40 (22)	29 (22)	0	8 (100)	1 (25)	0	1 (33)	1 (50)	0	0
ESBL+, no. (%)	26 (14)	25 (19)	0	0	1 (25)	0	0	0	0	0
Susceptibility rate, %										
Ampicillin	48	62	0	0	50	0	0	0	50	0
Ampicillin-sulbactam	65	70	82	0	75	0	0	0	100	50
Piperacillin-tazobactam	98	100	96	88	100	100	67	100	100	100
Cefazolin	62	70	73	0	25	0	0	0	50	0
Cefmetazole	87	96	86	0	100	0	67	0	100	50
Ceftriaxone	78	78	100	0	75	100	67	50	100	100
Cefepime	83	79	100	88	75	100	100	100	100	100
Meropenem	98	100	100	63	100	100	100	100	100	100
Aztreonam	79	79	96	63	75	100	33	50	100	0
Amikacin	100	100	100	100	100	100	100	100	100	100
Levofloxacin	76	68	100	88	100	100	100	100	100	100
TMP-SMX	85	90	91	0	50	100	100	50	100	100
Minocycline	83	91	86	0	0	100	33	50	100	100

Abbreviations: GNR, Gram-negative rods; ESBL, extended-spectrum β-lactamase; TMP-SMX, trimethoprim-sulfamethoxazole.

**Table 3:** **Antibiotic** susceptibility pattern of isolates from nursing home residents and community-dwelling patients.

Antibiotics	Overall		Patients Living in Nursing Home		Community-Dwelling Patients	
	Enterobacterales (*n* = 171)	*Pseudomonas* *aeruginosa* (*n* = 8)	Enterobacterales (*n* = 39)	*Pseudomonas* *aeruginosa* (*n* = 3)	Enterobacterales (*n* = 132)	*Pseudomonas* *aeruginosa* (*n* = 5)
Susceptibility rate, %						
Ampicillin	50	0	36	0	55	0
Ampicillin-sulbactam	68	0	59	0	71	0
Piperacillin-tazobactam	99	88	100	100	99	80
Cefazolin	66	0	56	0	69	0
Cefmetazole	92	0	90	0	93	0
Ceftriaxone	81	0	64	0	86	0
Cefepime	83	88	69	100	87	80
Meropenem	100	63	100	100	100	40
Aztreonam	81	63	67	33	85	80
Amikacin	100	100	100	100	100	100
Levofloxacin	75	88	54	100	81	80
TMP-SMX	89	0	79	0	92	0
Minocycline	87	0	82	0	87	0

Abbreviations: TMP-SMX, trimethoprim-sulfamethoxazole.

**Table 4:** Multivariate analysis for resistant Gram-negative rods.

Risk Factors	Odds Ratio (95% CI)	*p*-Value
Age	1.00 (0.96–1.03)	0.900
Nursing home residence	2.83 (1.18–6.79)	0.020
Antibiotic use within 3 months	4.52 (1.02–19.97)	0.047
Long-term urinary catheter placement	2.77 (0.81–9.45)	0.103

Abbreviation: CI, confidence interval. Significant values are indicated in bold.
